# Supplementary material for: Prevalence and Prognostic Significance of Malnutrition in Patients with Abnormal Glycemic Status and Coronary Artery Disease: A Multicenter Cohort Study in China
Source: Nutrients. 2023 Feb 1;15(3):732. doi: 10.3390/nu15030732 (PMC9920677; doi:10.3390/nu15030732)
Supplement: Supplementary file 1 [file nutrients-15-00732-s001.zip › nutrients-2143085-supplementary.pdf]

Supplementary Materials for

Prevalence and prognostic significance of malnutrition in patients with abnormal glycemic status and coronary artery disease: a multicenter cohort study in China

Table S1 Baseline characteristics of patients died before discharge and lost to follow-up 2
Table S2 Baseline characteristics of patients grouped by glycemic status 4
Table S3 Baseline characteristics of matched patients with and without malnutrition according to the GLIM criteria 6
Table S4 Baseline characteristics of matched patients with and without malnutrition according to PNI 7
Table S5 Baseline characteristics of matched patients with different nutritional statuses according to the COUNT score 8
Table S6 Baseline characteristics of matched patients with different nutritional statuses according to NRI 9
Table S7 Proportion of malnutrition according to each assessment tool stratified by age group, sex and presentation of coronary artery disease 10
Table S8 Performance of four nutritional assessment tools for predicting all-cause death grouped by glycemic status 11
Table S9 Performance of four nutritional assessment tools for predicting MACCEs grouped by glycemic status 12
Figure S1 Study flowchart 13
Figure S2 Incidence of MACCEs 14
Figure S3 Potential interaction of glycemic status and nutritional status on predicting all-cause death 15
Figure S4 Potential interaction of glycemic status and nutritional status on predicting MACCEs 16
Figure S5 Subgroup analysis of associations of nutritional status with all-cause death 17
Figure S6 Subgroup analysis of associations of nutritional status with MACCEs 18
Figure S7 RCSs of PNI, COUNT and NRI for predicting MACCEs 19

Table S1 Baseline characteristics of patients died before discharge and lost to follow-up

	Died before discharge (n=75)	p	Lost to follow-up (n=194)	p
**Demographic characteristics**				
Age, years	68 [63-77]	<0.001	64 [57-69]	0.009
Female	34 (45.33)	<0.001	53 (27.32)	0.669
Body mass index, kg/m^2	24.49 [22.85-27.04]	0.004	25 [24-28]	0.637
Smoking status				
Current smoker	20 (26.67)		52 (26.80)	
Former smoker	19 (25.33)	0.394	58 (29.90)	0.633
Never smoker	36 (48.00)		84 (43.30)	
**Clinical characteristics**				
CAD presentation				
ACS	72 (96.00)	<0.001	97 (50.00)	0.669
CCS	3 (4.00)		97 (50.00)	
Length of stay, day	5 [2-12]	0.262	5 [3-8]	0.750
Glycemic status				
Prediabetes	12 (16.00)	<0.001	77 (39.69)	0.623
Diabetes	63 (84.00)		117 (60.31)	
Hypertension	55 (73.33)	0.818	144 (74.23)	0.514
Dyslipidemia	58 (77.33)	<0.001	168 (86.60)	0.002
Peripheral artery disease	1 (1.33)	0.116	14 (7.22)	0.278
COPD	2 (2.67)	0.471	4 (2.06)	0.618
Prior myocardial infarction	13 (17.33)	0.951	41 (21.13)	0.194
Prior stroke	17 (22.67)	0.084	31 (15.98)	0.834
**Laboratory tests**				
FBG, mmol/L	10.10 [7.69-13.15]	<0.001	6.12 [5.12-7.92]	0.097
HbA1c, %	6.1 [5.8-7.3]	0.973	6.1 [6.0-7.1]	0.160
Lymphocyte count, ×10^9/L	1.70 [1.08-14.75]	0.602	1.97 [1.48-12.13]	0.008
Serum albumin, g/L	37.1 [33.2-39.2]	<0.001	42.6 [38.8-46.0]	0.915
hs-CRP, mg/L	7.72 [1.92-12.30]	<0.001	1.92 [0.86-3.84]	0.140
Total cholesterol, mmol/L	3.97 [3.40-4.71]	0.513	3.91 [3.29-4.84]	0.525
eGFR <60 ml/min/1.73m^2	19 (25.33)	0.001	6 (3.09)	0.921
LVEF <40%	22 (29.33)	<0.001	11 (5.67)	0.021
**Angiographic characteristics**				
Coronary angiography	60 (80.00)	<0.001	182 (93.81)	<0.001
LMCA/three-vessel disease	53 (70.67)	<0.001	93 (47.94)	0.802
SYNTAX score				
≤22	33 (55.00)		147 (80.77)	
23-32	15 (25.00)	<0.001	26 (14.29)	0.739
≥33	12 (20.00)		9 (4.95)	
PCI	47 (62.67)	0.057	73 (37.63)	<0.001
**Medication**				
Aspirin	55 (73.33)	0.001	189 (97.42)	0.162
$P2Y_{12}$ inhibitors	44 (58.67)	<0.001	148 (76.29)	<0.001
Statins	33 (44.00)	<0.001	188 (96.91)	0.439
β-blockers	28 (37.3)	<0.001	155 (79.90)	0.701
ACEIs/ARBs	21 (28.00)	<0.001	118 (60.82)	0.614
**Malnutrition**				
GLIM	19 (25.33)	<0.001	16 (8.25)	0.989
PNI	33 (44.00)	<0.001	14 (7.22)	0.735
COUNT	52 (69.34)	<0.001	122 (62.89)	0.179
Mild	38 (50.67)		117 (60.31)	
Moderate-severe	14 (18.67)		5 (2.58)	
NRI	43 (57.34)	<0.001	40 (20.61)	0.682
Mild	11 (14.67)		17 (8.76)	
Moderate-severe	32 (42.67)		23 (11.85)	

p-value indicates the comparison with all participants included in the final analysis.

Values are presented as number (%) or median [interquartile range].

ACEI, angiotensin-converting enzyme inhibitor; ACS, acute coronary syndrome; ARB, angiotensin-receptor blocker; CCS, chronic coronary syndrome; COPD, chronic obstructive pulmonary disease; COUNT, controlling

nutritional status; eGFR, estimated glomerular filtration rate; FBG, fasting blood glucose; GLIM, global leadership initiative on malnutrition; HbA1c, glycated hemoglobin; hs-CRP, high-sensitivity C-reactive protein; LMCA, left main coronary artery; LVEF, left ventricular ejection fraction; NRI, nutritional risk index; PCI, percutaneous coronary intervention; PNI, prognostic nutritional index; SYNTAX, synergy between percutaneous coronary intervention with Taxus and cardiac surgery.

Table S2 Baseline characteristics of patients grouped by glycemic status

	Prediabetes (n=5710)	Diabetes (n=9328)	p
**Demographic characteristics**			
Age, years	62 [54-68]	62 [55-67]	0.064
Female	1406 (24.62)	2498 (26.78)	0.003
Body mass index, kg/m^2	25.39 [23.44-27.55]	25.93 [23.88-28.04]	<0.001
Smoking status			
Current smoker	1500 (26.27)	2174 (23.31)	
Former smoker	1804 (31.59)	3124 (33.49)	<0.001
Never smoker	2406 (42.14)	4030 (43.20)	
**Clinical characteristics**			
CAD presentation			<0.001
ACS	2541 (44.50)	4742 (50.84)	
CCS	3169 (55.50)	4586 (49.16)	
Length of stay, day	5 [3-7]	5 [3-8]	<0.001
Hypertension	3818 (66.87)	7025 (75.31)	<0.001
Dyslipidemia	5140 (90.02)	8763 (93.94)	<0.001
Peripheral artery disease	268 (4.69)	548 (5.87)	0.002
COPD	103 (1.80)	139 (1.49)	0.138
Prior myocardial infarction	929 (16.27)	1713 (18.36)	0.001
Prior stroke	776 (13.59)	1543 (16.54)	<0.001
**Laboratory tests**			
FBG, mmol/L	5.47 [4.96-5.98]	6.80 [6.00-7.80]	<0.001
HbA1c, %	5.9 [5.7-6.1]	6.8 [6.0-7.8]	<0.001
Lymphocyte count, ×10^9/L	1.90 [1.41-2.73]	1.81 [1.35-2.48]	<0.001
Serum albumin, g/L	43.20 [39.6-46.6]	42.0 [38.6-45.8]	<0.001
hs-CRP, mg/L	1.92 [0.93-3.71]	1.98 [1.09-5.51]	<0.001
Total cholesterol, mmol/L	4.00 (3.40-4.76)	3.99 (3.35-4.75)	0.119
eGFR <60 ml/min/1.73m^2	105 (1.84)	333 (3.57)	<0.001
LVEF <40%	120 (2.10)	311 (3.33)	<0.001
**Angiographic characteristics**			
Coronary angiography	5626 (98.53)	9190 (98.52)	0.995
LMCA/three-vessel disease	2368 (41.47)	4703 (50.42)	<0.001
SYNTAX score			<0.001
≤22	4741 (84.30)	7513 (81.81)	
23-32	680 (12.09)	1278 (13.92)	
≥33	204 (3.61)	393 (4.28)	
PCI	4089 (71.61)	6881 (73.77)	0.004
**Medication**			
Aspirin	5633 (98.65)	9196 (98.58)	0.735
$P2Y_{12}$ inhibitors	5053 (88.49)	8477 (90.88)	<0.001
Statins	5577 (97.67)	9123 (97.80)	0.597
β-blockers	4479 (78.44)	7704 (82.59)	<0.001
ACEIs/ARBs	3293 (57.67)	6124 (65.65)	<0.001
**Malnutrition**			
GLIM	502 (8.79)	974 (10.44)	<0.001
PNI	327 (5.73)	856 (9.18)	<0.001
COUNT	3149 (55.15)	5411 (58.00)	<0.001
Mild	2997 (52.49)	5071 (54.36)	
Moderate-severe	152 (2.66)	340 (3.64)	
NRI	1128 (19.75)	1910 (20.48)	0.348
Mild	453 (7.93)	802 (8.60)	
Moderate-severe	675 (11.82)	1108 (11.88)	

Values are presented as number (%) or median [interquartile range].

ACEI, angiotensin-converting enzyme inhibitor; ACS, acute coronary syndrome; ARB, angiotensin-receptor blocker; CCS, chronic coronary syndrome; COPD, chronic obstructive pulmonary disease; COUNT, controlling nutritional status; eGFR, estimated glomerular filtration rate; FBG, fasting blood glucose; GLIM, global leadership initiative on malnutrition; HbA1c, glycated hemoglobin; hs-CRP, high-sensitivity C-reactive protein; LMCA, left main coronary artery; LVEF, left ventricular ejection fraction; NRI, nutritional risk index; PCI, percutaneous coronary intervention; PNI, prognostic nutritional index; SYNTAX, synergy between percutaneous coronary intervention with Taxus and cardiac surgery.

Table S3 Baseline characteristics of matched patients with and without malnutrition according to the GLIM criteria

	Malnutrition (n=407)	Non-malnutrition (n=1628)	p
**Demographic characteristics**			
Age, years	68 [62-76]	66 (59-71)	<0.001
Female	188 (39.25)	650 (33.92)	0.029
Body mass index, kg/m^2	23.15 [22.04-24.24]	24.24 [22.86-25.78]	<0.001
Smoking status			
Current smoker	77 (18.92)	332 (20.39)	
Former smoker	125 (30.71)	513 (31.51)	<0.001
Never smoker	205 (50.37)	783 (48.96)	
**Clinical characteristics**			
CAD presentation			
ACS	214 (44.68)	1081 (56.42)	<0.001
CCS	265 (55.32)	835 (43.58)	
Length of stay, day	5 [3-9]	5 [3-8]	0.192
Glycemic status			
Prediabetes	187 (39.04)	760 (39.67)	0.802
Diabetes	292 (60.96)	1156 (60.33)	
Hypertension	340 (70.98)	1392 (72.65)	0.465
Dyslipidemia	429 (89.56)	1789 (93.37)	0.004
Peripheral artery disease	33 (6.89)	129 (6.73)	0.903
COPD	7 (1.46)	34 (1.77)	0.637
Prior myocardial infarction	76 (15.87)	330 (17.22)	0.479
Prior stroke	83 (17.33)	350 (18.27)	0.633
**Laboratory tests**			
FBG, mmol/L	6.33 [5.31-8.04]	6.23 [5.32-8.09]	0.608
HbA1c, %	6.1 [5.8-7.1]	6.1 [5.8-7.0]	0.861
Lymphocyte count, ×10^9/L	1.71 [1.32-2.24]	1.80 [1.31-2.66]	0.011
Serum albumin, g/L	41.3 [37.3-44.8]	42.0 [38.6-45.6]	<0.001
hs-CRP, mg/L	7.03 [4.22-10.89]	1.92 [0.86-3.55]	<0.001
Total cholesterol, mmol/L	4.11 [3.42-4.84]	4.00 [3.38-4.71]	0.152
eGFR <60 ml/min/1.73m^2	13 (2.71)	77 (4.02)	0.179
LVEF <40%	12 (2.51)	68 (3.55)	0.255
**Angiographic characteristics**			
Coronary angiography	473 (98.75)	1875 (97.86)	0.210
LMCA/three-vessel disease	202 (42.17)	973 (50.78)	<0.001
SYNTAX score			
≤22	376 (79.49)	1513 (80.69)	
23-32	72 (15.22)	264 (14.08)	0.813
≥33	25 (5.29)	98 (5.23)	
PCI	339 (70.77)	1429 (74.58)	0.090
**Medication**			
Aspirin	430 (89.77)	1731 (90.34)	0.567
$P2Y_{12}$ inhibitors	430 (89.77)	1731 (90.34)	0.705
Statins	469 (97.91)	1871 (97.65)	0.733
β-blockers	405 (84.55)	1512 (78.91)	0.006
ACEIs/ARBs	283 (59.08)	1214 (63.36)	0.084

Values are presented as number (%) or median [interquartile range].
ACEI, angiotensin-converting enzyme inhibitor; ACS, acute coronary syndrome; ARB, angiotensin-receptor blocker; CCS, chronic coronary syndrome; COPD, chronic obstructive pulmonary disease; eGFR, estimated glomerular filtration rate; FBG, fasting blood glucose; GLIM, global leadership initiative on malnutrition; HbA1c, glycated hemoglobin; hs-CRP, high-sensitivity C-reactive protein; LMCA, left main coronary artery; LVEF, left ventricular ejection fraction; PCI, percutaneous coronary intervention; SYNTAX, synergy between percutaneous coronary intervention with Taxus and cardiac surgery.

Table S4 Baseline characteristics of matched patients with and without malnutrition according to PNI

	Malnutrition (n=1051)	Non-malnutrition (n=4204)	p
**Demographic characteristics**			
Age, years	66 [60,74]	65 [56-71]	<0.001
Female	297 (28.26)	1145 (27.24)	0.506
Body mass index, kg/m^2	24.91 [22.95-27.04]	25.39 [23.38-27.76]	<0.001
Smoking status			
Current smoker	241 (22.93)	1150 (27.36)	
Former smoker	330 (31.40)	1228 (29.21)	<0.001
Never smoker	480 (45.67)	1826 (43.44)	
**Clinical characteristics**			
CAD presentation			
ACS	864 (82.21)	3128 (74.41)	<0.001
CCS	187 (17.79)	1076 (25.59)	
Length of stay, day	7 [5-10]	6 [4-9]	<0.001
Glycemic status			
Prediabetes	305 (32.83)	1525 (36.27)	0.037
Diabetes	706 (67.17)	2679 (63.73)	
Hypertension	748 (71.17)	2922 (69.51)	0.293
Dyslipidemia	967 (92.01)	3776 (89.82)	0.032
Peripheral artery disease	45 (4.28)	211 (5.02)	0.321
COPD	37 (3.52)	63 (1.50)	<0.001
Prior myocardial infarction	175 (16.65)	690 (16.41)	0.852
Prior stroke	179 (17.03)	634 (15.08)	0.118
**Laboratory tests**			
FBG, mmol/L	7.30 [5.98-9.50]	6.61 [5.56-8.51]	<0.001
HbA1c, %	6.0 [5.7-7.0]	6.1 [5.8-7.1]	0.003
Lymphocyte count, $\times 10^9$/L	1.15 [0.88-1.62]	1.99 [1.46-3.48]	<0.001
Serum albumin, g/L	35.0 [33.3-36.5]	41.4 [38.8-45.0]	<0.001
hs-CRP, mg/L	5.11 [1.92-11.32]	1.97 [1.30-5.55]	<0.001
Total cholesterol, mmol/L	3.87 [3.26-4.51]	4.08 [3.43-4.82]	<0.001
eGFR <60 ml/min/1.73m^2	44 (4.19)	100 (2.38)	0.001
LVEF <40%	48 (4.57)	158 (3.76)	0.227
**Angiographic characteristics**			
Coronary angiography	1027 (97.72)	4103 (97.60)	0.821
LMCA/three-vessel disease	554 (52.71)	2021 (48.07)	0.007
SYNTAX score			
≤22	801 (78.07)	3309 (80.69)	
23-32	163 (15.89)	580 (14.14)	0.166
≥33	62 (6.04)	212 (5.17)	
PCI	841 (80.02)	3193 (75.95)	0.005
**Medication**			
Aspirin	1035 (98.48)	4146 (98.62)	0.725
$P2Y_{12}$ inhibitors	972 (92.48)	3834 (91.20)	0.183
Statins	1024 (97.43)	4092 (97.34)	0.863
β-blockers	836 (79.54)	3228 (76.78)	0.056
ACEIs/ARBs	706 (67.17)	2679 (63.73)	0.037

Values are presented as number (%) or median [interquartile range].
ACEI, angiotensin-converting enzyme inhibitor; ACS, acute coronary syndrome; ARB, angiotensin-receptor blocker; CCS, chronic coronary syndrome; COPD, chronic obstructive pulmonary disease; eGFR, estimated glomerular filtration rate; FBG, fasting blood glucose; HbA1c, glycated hemoglobin; hs-CRP, high-sensitivity C-reactive protein; LMCA, left main coronary artery; LVEF, left ventricular ejection fraction; PCI, percutaneous coronary intervention; PNI, prognostic nutritional index; SYNTAX, synergy between percutaneous coronary intervention with Taxus and cardiac surgery.

Table S5 Baseline characteristics of matched patients with different nutritional statuses according to the COUNT score

	Moderate-severe Malnutrition (n=492)	Mild malnutrition (n=1098)	Non-malnutrition (n=870)	p
**Demographic characteristics**				
Age, years	66 [59-74]	63 [56-69]	60 [53-66]	<0.001
Female	112 (22.76)	362 (32.97)	370 (42.53)	<0.001
Body mass index, kg/m^2	24.73 [22.57-27.02]	26.05 [23.75-29.41]	27.06 [24.34-30.12]	<0.001
Smoking status				
Current smoker	104 (21.14)	225 (20.49)	204 (23.45)	
Former smoker	173 (35.16)	346 (31.51)	237 (27.24)	<0.001
Never smoker	215 (43.70)	527 (48.00)	429 (49.31)	
**Clinical characteristics**				
CAD presentation				
ACS	307 (62.40)	450 (40.98)	349 (40.11)	<0.001
CCS	185 (37.60)	648 (59.02)	521 (59.89)	
Length of stay, day	6 [4-10]	5 [3-8]	5 [3-7]	<0.001
Glycemic status				
Prediabetes	152 (30.89)	401 (36.52)	364 (41.84)	<0.001
Diabetes	340 (69.11)	697 (63.48)	506 (58.16)	
Hypertension	334 (67.89)	844 (76.87-76.90)	669 (76.90)	<0.001
Dyslipidemia	464 (94.31)	968 (88.16)	753 (86.55)	<0.001
Peripheral artery disease	26 (5.28)	67 (6.10)	37 (4.25)	0.191
COPD	20 (4.07)	22 (2.00)	11 (1.26)	0.003
Prior myocardial infarction	106 (21.54)	209 (19.03)	93 (10.69)	<0.001
Prior stroke	79 (16.06)	193 (17.58)	138 (15.86)	0.551
**Laboratory tests**				
FBG, mmol/L	6.60 [5.42-8.48]	6.24 [5.32-7.94]	6.26 [5.36-8.04]	0.133
HbA1c, %	6.0 [5.7-6.9]	6.2 [5.9-7.2]	6.2 [5.8-7.3]	<0.001
Lymphocyte count, $\times 10^9$/L	0.96 [0.69-1.44]	1.53 [1.19-2.21]	2.12 [1.79-2.79]	<0.001
Serum albumin, g/L	35.5 [32.5-41.6]	42.3 [38.5-46.0]	43.4 [39.9-46.8]	<0.001
hs-CRP, mg/L	2.82 [1.55-10.71]	1.92 [0.92-4.19]	1.98 [1.33-5.08]	<0.001
Total cholesterol, mmol/L	3.08 (2.54-3.50)	3.49 (3.12-4.10)	4.72 (4.19-5.36)	<0.001
eGFR <60 ml/min/1.73m^2	33 (6.71)	50 (4.55)	26 (2.99)	0.006
LVEF <40%	29 (5.89)	42 (3.83)	23 (2.64)	0.011
**Angiographic characteristics**				
Coronary angiography	471 (95.73)	1079 (47.81)	394 (45.29)	0.004
LMCA/three-vessel disease	248 (50.41)	525 (47.81)	394 (4.29)	0.181
SYNTAX score				
≤22	373 (79.19)	896 (83.04)	710 (83.14)	
23-32	65 (13.80)	127 (11.77)	116 (13.58)	0.022
≥33	33 (7.01)	56 (5.19)	28 (3.28)	
PCI	356 (72.36)	789 (71.86)	670 (77.01)	0.026
**Medication**				
Aspirin	475 (96.54)	1078 (98.18)	858 (99.54)	0.701
$P2Y_{12}$ inhibitors	451 (91.67)	990 (90.16)	772 (88.74)	0.214
Statins	482 (97.97)	1065 (96.99)	849 (97.59)	0.483
β-blockers	385 (78.25)	920 (83.79)	708 (81.38)	0.027
ACEIs/ARBs	302 (61.38)	677 (61.66)	568 (65.29)	0.189

Values are presented as number (%) or median [interquartile range].
ACEI, angiotensin-converting enzyme inhibitor; ACS, acute coronary syndrome; ARB, angiotensin-receptor blocker; CCS, chronic coronary syndrome; COPD, chronic obstructive pulmonary disease; COUNT, controlling nutritional status; eGFR, estimated glomerular filtration rate; FBG, fasting blood glucose; HbA1c, glycated hemoglobin; hs-CRP, high-sensitivity C-reactive protein; LMCA, left main coronary artery; LVEF, left ventricular ejection fraction; PCI, percutaneous coronary intervention; SYNTAX, synergy between percutaneous coronary intervention with Taxus and cardiac surgery.

Table S6 Baseline characteristics of matched patients with different nutritional statuses according to NRI

	Moderate-severe Malnutrition (n=1232)	Mild malnutrition (n=493)	Non-malnutrition (n=4435)	p
**Demographic characteristics**				
Age, years	64 [57-71]	63 [57-69]	62 [54-68]	<0.001
Female	326 (26.46)	159 (32.25)	1101 (24.83)	0.001
Body mass index, kg/m^2	25.35 [23.51-27.44]	25.16 [23.44-27.34]	25.78 [23.79-28.04]	<0.001
Smoking status				
Current smoker	329 (26.71)	129 (26.17)	1067 (24.06)	
Former smoker	336 (27.27)	144 (29.21)	1513 (34.12)	
Never smoker	567 (46.02)	220 (44.63)	1855 (41.83)	
**Clinical characteristics**				
CAD presentation				
ACS	749 (60.80)	318 (64.50)	2225 (50.17)	<0.001
CCS	483 (39.20)	175 (35.50)	2210 (49.83)	
Length of stay, day	6 [3-9]	6 [3-9]	5 [3-8]	<0.001
Glycemic status				
Prediabetes	474 (38.47)	155 (31.44)	1680 (37.88)	0.014
Diabetes	758 (61.53)	338 (68.56)	2755 (62.12)	
Hypertension	871 (70.70)	345 (69.98)	3218 (72.56)	0.257
Dyslipidemia	1131 (91.80)	447 (90.67)	4123 (92.97)	0.098
Peripheral artery disease	58 (4.71)	22 (4.46)	262 (5.91)	0.145
COPD	17 (1.38)	4 (0.81)	71 (1.60)	0.365
Prior myocardial infarction	213 (17.29)	81 (16.43)	774 (17.45)	0.850
Prior stroke	196 (15.91)	63 (12.78)	731 (16.48)	0.103
**Laboratory tests**				
FBG, mmol/L	6.01 [5.02-8.14]	6.41 [5.13-8.21]	6.39 [5.45-8.11]	<0.001
HbA1c, %	6.1 [5.8-7.3]	6.1 [5.8-7.2]	6.2 [5.8-7.1]	0.485
Lymphocyte count, ×10^9/L	2.04 [1.41-13.70]	2.02 [1.37-13.40]	1.80 [1.36-2.43]	<0.001
Serum albumin, g/L	36.0 [34.3-36.9]	38.3 [37.8-38.9]	43.6 [40.7-46.9]	<0.001
hs-CRP, mg/L	2.35 [1.63-9.62]	1.92 [1.16-6.29]	1.92 [0.98-4.34]	<0.001
Total cholesterol, mmol/L	3.83 [3.21-4.55]	3.80 [3.16-4.74]	4.07 [3.39-4.81]	<0.001
eGFR <60 ml/min/1.73m^2	42 (3.41)	14 (2.84)	89 (2.01)	0.012
LVEF <40%	44 (3.57)	9 (1.83)	121 (2.72)	0.108
**Angiographic characteristics**				
Coronary angiography	1212 (98.38)	477 (96.75)	4379 (98.74)	0.002
LMCA/three-vessel disease	607 (49.27)	261 (52.94)	2032 (45.82)	0.002
SYNTAX score				
≤22	938 (77.46)	368 (77.31)	3646 (83.28)	<0.001
23-32	194 (16.02)	75 (15.76)	582 (13.29)	
≥33	79 (6.52)	33 (6.93)	150 (3.43)	
PCI	941 (76.38)	366 (74.24)	3185 (71.82)	0.005
**Medication**				
Aspirin	1216 (98.70)	485 (98.38)	4373 (98.60)	0.874
$P2Y_{12}$ inhibitors	1127 (91.48)	448 (90.87)	3952 (89.11)	0.036
Statins	1200 (97.40)	478 (96.96)	4346 (97.99)	0.193
β-blockers	883 (71.67)	353 (71.60)	3682 (83.02)	<0.001
ACEIs/ARBs	805 (65.34)	334 (67.75)	2803 (63.20)	0.074

Values are presented as number (%) or median [interquartile range].
ACEI, angiotensin-converting enzyme inhibitor; ACS, acute coronary syndrome; ARB, angiotensin-receptor blocker; CCS, chronic coronary syndrome; COPD, chronic obstructive pulmonary disease; eGFR, estimated glomerular filtration rate; FBG, fasting blood glucose; HbA1c, glycated hemoglobin; hs-CRP, high-sensitivity C-reactive protein; LMCA, left main coronary artery; LVEF, left ventricular ejection fraction; NRI, nutritional risk index; PCI, percutaneous coronary intervention; SYNTAX, synergy between percutaneous coronary intervention with Taxus and cardiac surgery.

Table S7 Proportion of malnutrition according to each assessment tool stratified by age group, sex and presentation of coronary artery disease

Nutritional assessment tool	Prediabetes (n=5710)			Diabetes (n=9328)		
	**Age<65 (n=3535)**	**≥65 (n=2175)**	**p**	**<65 (n=5730)**	**≥65 (n=3598)**	**p**
GLIM	110 (3.11)	302 (13.89)	<0.001	179 (3.12)	652 (18.12)	<0.001
PNI	121 (3.42)	206 (9.47)	<0.001	335 (5.85)	521 (14.48)	<0.001
COUNT			<0.001			<0.001
Mild	1800 (50.92)	1197 (55.03)		3037 (53.00)	2034 (56.53)	
Moderate-severe	66 (1.87)	86 (3.95)		147 (2.57)	193 (5.36)	
NRI			<0.001			<0.001
Mild	244 (6.90)	209 (9.61)		422 (7.36)	380 (10.56)	
Moderate-severe	297 (8.40)	378 (17.38)		445 (7.77)	663 (18.43)	
	**Male (n=4304)**	**Female (n=1406)**	**p**	**Male (n=6830)**	**Female (2498)**	**p**
GLIM	238 (5.53)	174 (12.38)	<0.001	377 (5.52)	454 (18.17)	<0.001
PNI	262 (6.09)	65 (4.62)	0.040	569 (8.33)	287 (11.49)	<0.001
COUNT			<0.001			<0.001
Mild	2361 (54.86)	636 (45.23)		3877 (56.76)	1194 (47.80)	
Moderate-severe	130 (3.02)	22 (1.56)		250 (3.66)	90 (3.60)	
NRI			0.050			<0.001
Mild	324 (7.53)	129 (9.17)		543 (7.95)	259 (10.37)	
Moderate-severe	526 (12.22)	149 (10.60)		733 (10.73)	375 (15.01)	
	**CCS (n=3169)**	**ACS (n=2541)**	**p**	**CCS (n=4586)**	**ACS (n=4742)**	**p**
GLIM	163 (5.14)	249 (9.80)	<0.001	215 (4.69)	616 (12.99)	<0.001
PNI	70 (2.21)	257 (10.11)	<0.001	117 (2.55)	739 (15.58)	<0.001
COUNT			<0.001			<0.001
Mild	1742 (54.97)	1255 (49.39)		2586 (56.39)	2485 (52.40)	
Moderate-severe	59 (1.86)	93 (3.66)		126 (2.75)	214 (4.51)	
NRI			<0.001			<0.001
Mild	192 (6.06)	261 (10.27)		313 (6.83)	489 (10.31)	
Moderate-severe	210 (6.63)	465 (18.30)		302 (6.59)	806 (17.00)	

Values are presented as number (%).
ACS, acute coronary syndrome; CCS, chronic coronary syndrome; COUNT, controlling nutritional status; GLIM, global leadership initiative on malnutrition; NRI, nutritional risk index; PNI, prognostic nutritional index.

Table S8 Performance of four nutritional assessment tools for predicting all-cause death grouped by glycemic status

	Prediabetes			
	**GLIM**	**PNI**	**COUNT**	**NRI**
**C-statistic (95% CI)**	0.58 (0.54, 0.62)	0.61 (0.55, 0.67)	0.62 (0.56, 0.67)	0.64 (0.60, 0.70)
**Comparison**				
PNI	cNRI: -0.153<br>IDI: -0.005	-	-	-
COUNT	cNRI: 0.015<br>IDI: -0.002	cNRI: 0.160<br>IDI: 0.003	-	-
NRI	cNRI: 0.083<br>IDI: 0.004	cNRI: 0.275***<br>IDI: 0.008***	cNRI: 0.130*<br>IDI: 0.006*	**-**
	**Diabetes**			
	**GLIM**	**PNI**	**COUNT**	**NRI**
**C-statistic (95% CI)**	0.61 (0.58, 0.64)	0.65 (0.62,0.68)	0.56 (0.53, 0.59)	0.64 (0.61, 0.67)
**Comparison**				
PNI	cNRI: -0.039<br>IDI: -0.007*	-	-	-
COUNT	cNRI: -0.230**<br>IDI: -0.013***	cNRI: -0.233**<br>IDI: -0.006**	-	-
NRI	cNRI: -0.021<br>IDI: -0.004	cNRI: 0.017<br>IDI: 0.002	cNRI: 0.168***<br>IDI: 0.009***	-

*p<0.05; ** p<0.01; ***p<0.001.

CI, confidence interval; cNRI, continuous net reclassification improvement; COUNT, controlling nutritional status; GLIM, global leadership initiative on malnutrition; IDI, integrated discrimination improvement; NRI, nutritional risk index; PNI, prognostic nutritional index.

Table S9 Performance of four nutritional assessment tools for predicting MACCEs grouped by glycemic status

	Prediabetes			
	**GLIM**	**PNI**	**COUNT**	**NRI**
**C-statistic (95% CI)**	0.53 (0.51, 0.55)	0.55 (0.51, 0.59)	0.56 (0.53, 0.60)	0.56 (0.52, 0.60)
**Comparison**				
PNI	cNRI: -0.033<br>IDI: -0.001	-	-	-
COUNT	cNRI: 0.034<br>IDI: 0.001	cNRI: 0.048<br>IDI: 0.001	-	-
NRI	cNRI: 0.028<br>IDI: 0.002	cNRI: 0.098<br>IDI: 0.003	cNRI: 0.037<br>IDI: 0.001	-
	**Diabetes**			
	**GLIM**	**PNI**	**COUNT**	**NRI**
**C-statistic (95% CI)**	0.56 (0.54, 0.57)	0.59 (0.57, 0.62)	0.53 (0.51, 0.56)	0.60 (0.57, 0.62)
**Comparison**				
PNI	cNRI: 0.012<br>IDI: -0.002	-	-	-
COUNT	cNRI: -0.112**<br>IDI: -0.007***	cNRI: -0.142***<br>IDI: -0.005***	-	-
NRI	cNRI: 0.033<br>IDI: -0.001	cNRI: 0.048<br>IDI: 0.002	cNRI: 0.127***<br>IDI: 0.007***	-

*p<0.05; ** p<0.01; ***p<0.001.

CI, confidence interval; cNRI, continuous net reclassification improvement; COUNT, controlling nutritional status; GLIM, global leadership initiative on malnutrition; IDI, integrated discrimination improvement; MACCE, major adverse cardiovascular and cerebrovascular event; NRI, nutritional risk index; PNI, prognostic nutritional index.

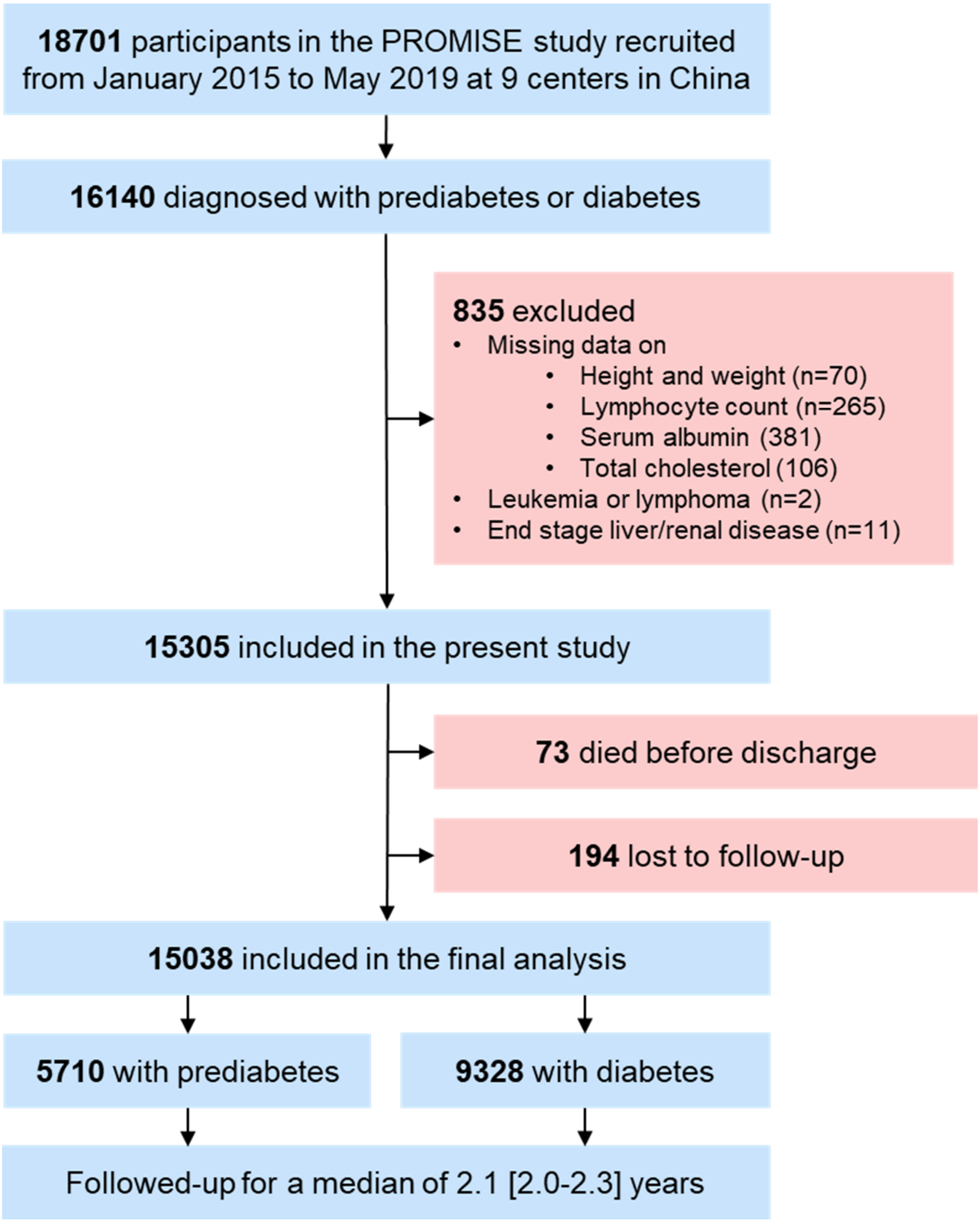

Figure S1 Study flowchart

PROMISE, PRospective Observational Multicenter cohort for ISchemic and hEmorrhage risk in coronary artery disease patients

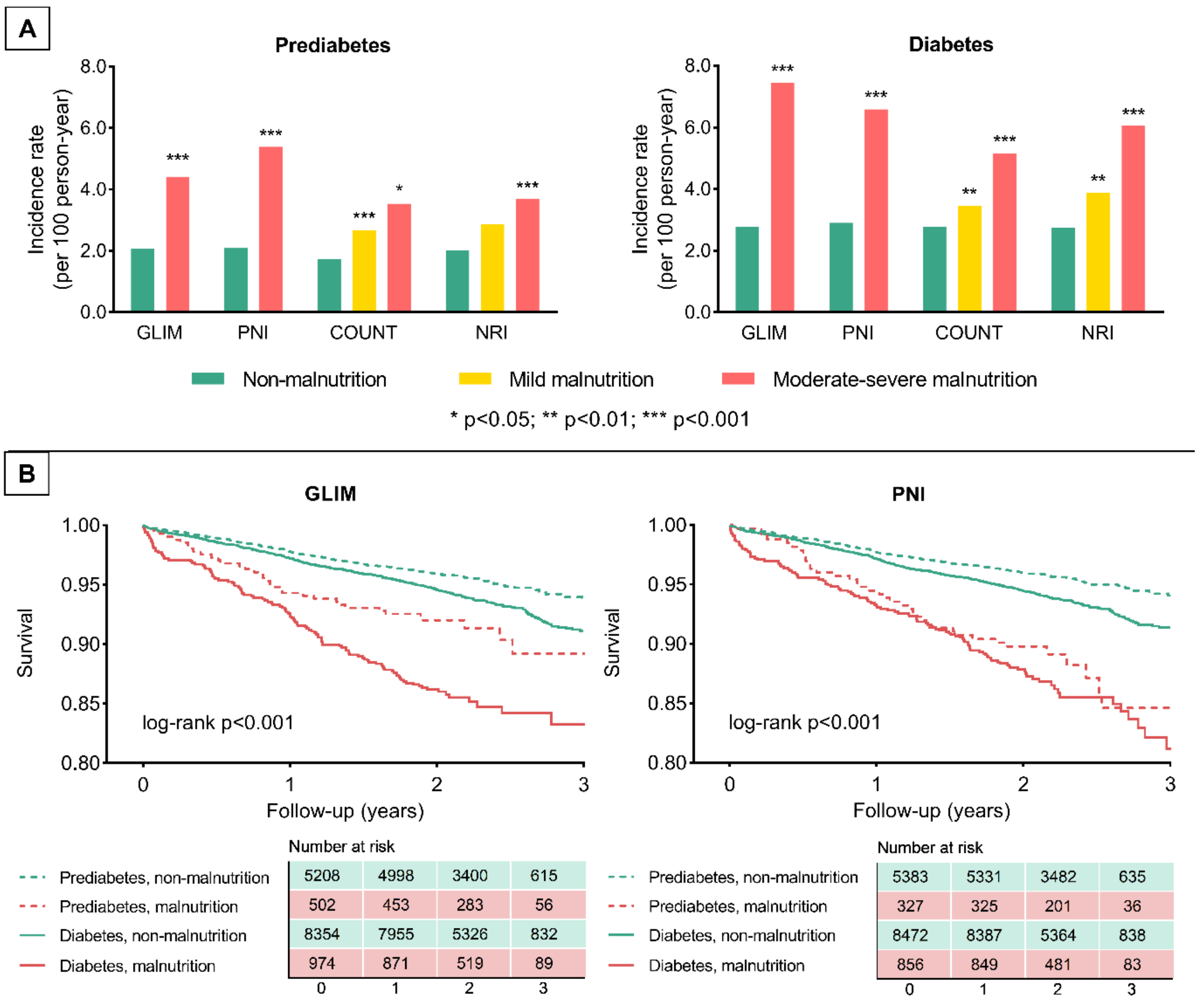

GLIM — Number at risk

	0	1	2	3
Prediabetes, non-malnutrition	5208	4998	3400	615
Prediabetes, malnutrition	502	453	283	56
Diabetes, non-malnutrition	8354	7955	5326	832
Diabetes, malnutrition	974	871	519	89

PNI — Number at risk

	0	1	2	3
Prediabetes, non-malnutrition	5383	5331	3482	635
Prediabetes, malnutrition	327	325	201	36
Diabetes, non-malnutrition	8472	8387	5364	838
Diabetes, malnutrition	856	849	481	83

COUNT — Number at risk

	0	1	2	3
Prediabetes, non-malnutrition	2561	2455	1670	301
Prediabetes, mild malnutrition	2997	2854	1915	345
Prediabetes, moderate-severe malnutrition	152	142	98	25
Diabetes, non-malnutrition	3917	3731	2508	385
Diabetes, mild malnutrition	5071	4790	3138	499
Diabetes, moderate-severe malnutrition	340	305	199	37

NRI — Number at risk

	0	1	2	3
Prediabetes, non-malnutrition	4582	4400	2957	502
Prediabetes, mild malnutrition	453	423	286	69
Prediabetes, moderate-severe malnutrition	675	628	440	100
Diabetes, non-malnutrition	7418	7058	4681	696
Diabetes, mild malnutrition	802	757	500	78
Diabetes, moderate-severe malnutrition	1108	1011	664	147

Figure S2 Incidence of MACCEs

(A) Incidence rate of MACCEs according to different assessment tools. **(B)** Event-free survival curves by glycemic status and nutritional status according to each assessment tool.

COUNT, Controlling Nutritional Status; GLIM, Global Leadership Initiative on Malnutrition; MACCE, major adverse cardiovascular and cerebrovascular event; NRI, nutritional risk index; PNI, prognostic nutritional index.

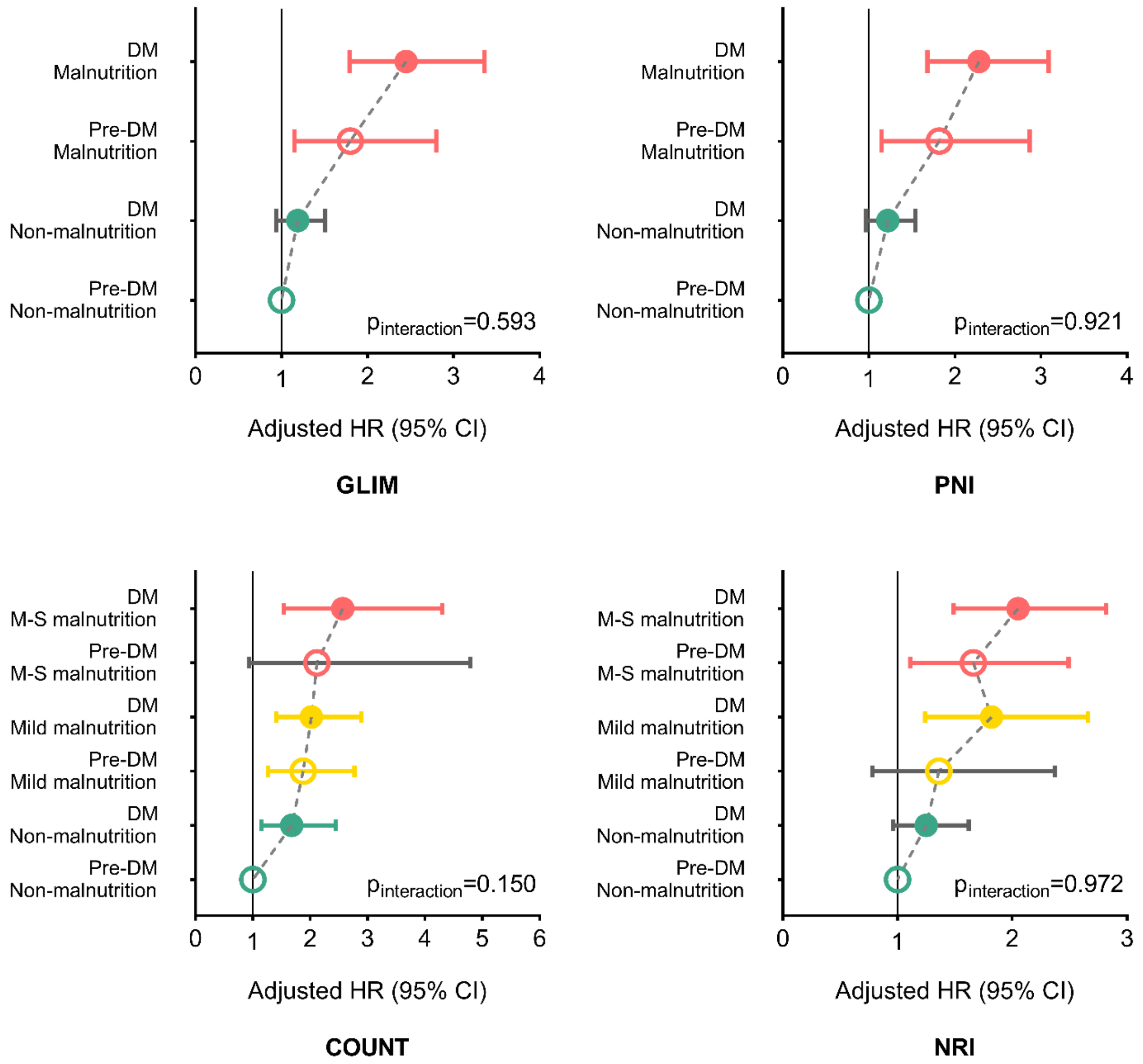

Figure S3 Potential interaction of glycemic status and nutritional status on predicting all-cause death

CI, confidence interval; COUNT, Controlling Nutritional Status; DM, diabetes; GLIM, Global Leadership Initiative on Malnutrition; HR, hazard ratio; M-S, moderate-severe; NRI, nutritional risk index; PNI, prognostic nutritional index.

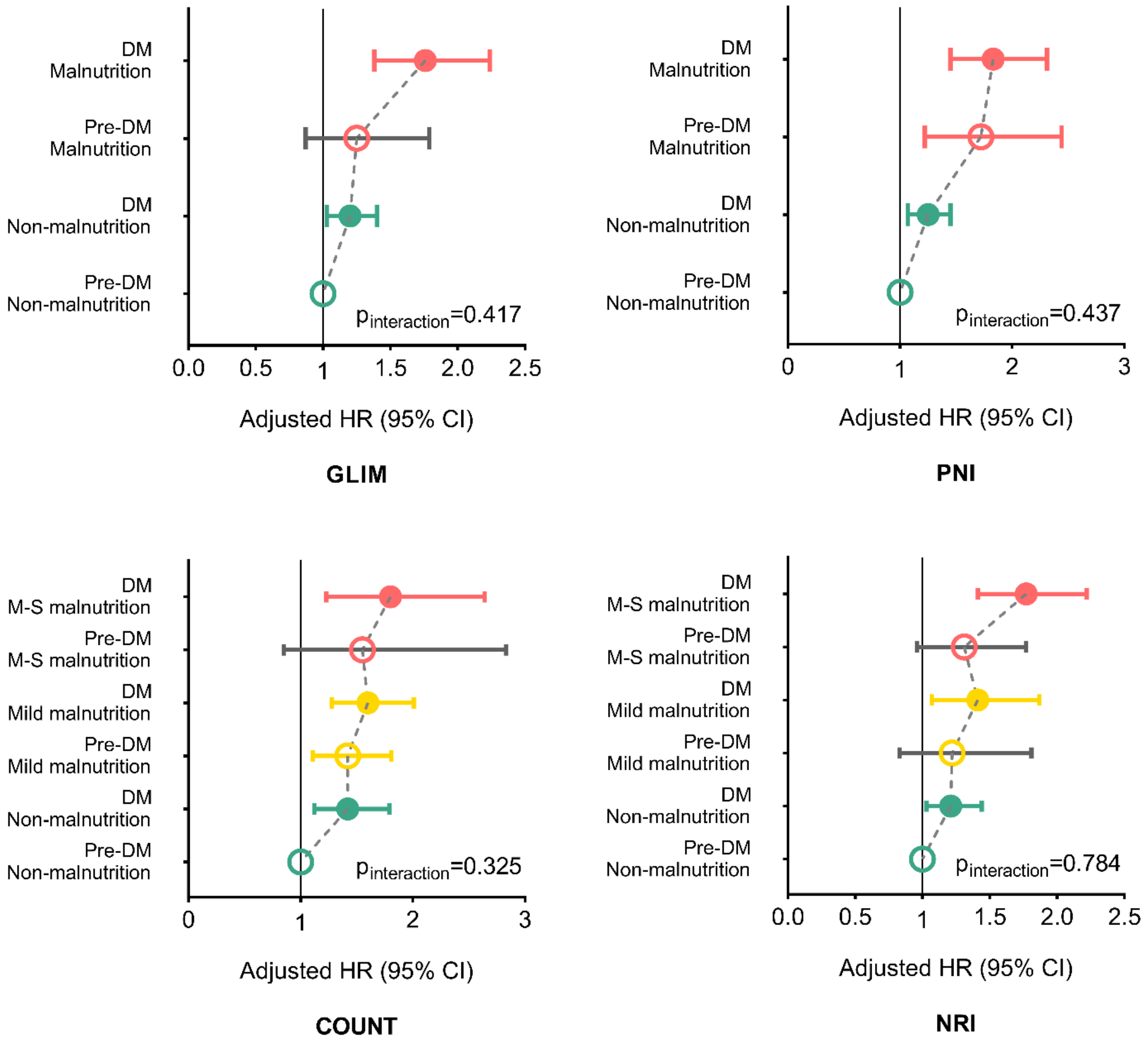

Figure S4 Potential interaction of glycemic status and nutritional status on predicting MACCEs
CI, confidence interval; COUNT, Controlling Nutritional Status; DM, diabetes; GLIM, Global Leadership Initiative on Malnutrition; HR, hazard ratio; MACCE, major adverse cardiovascular and cerebrovascular event; M-S, moderate-severe; NRI, nutritional risk index; PNI, prognostic nutritional index.

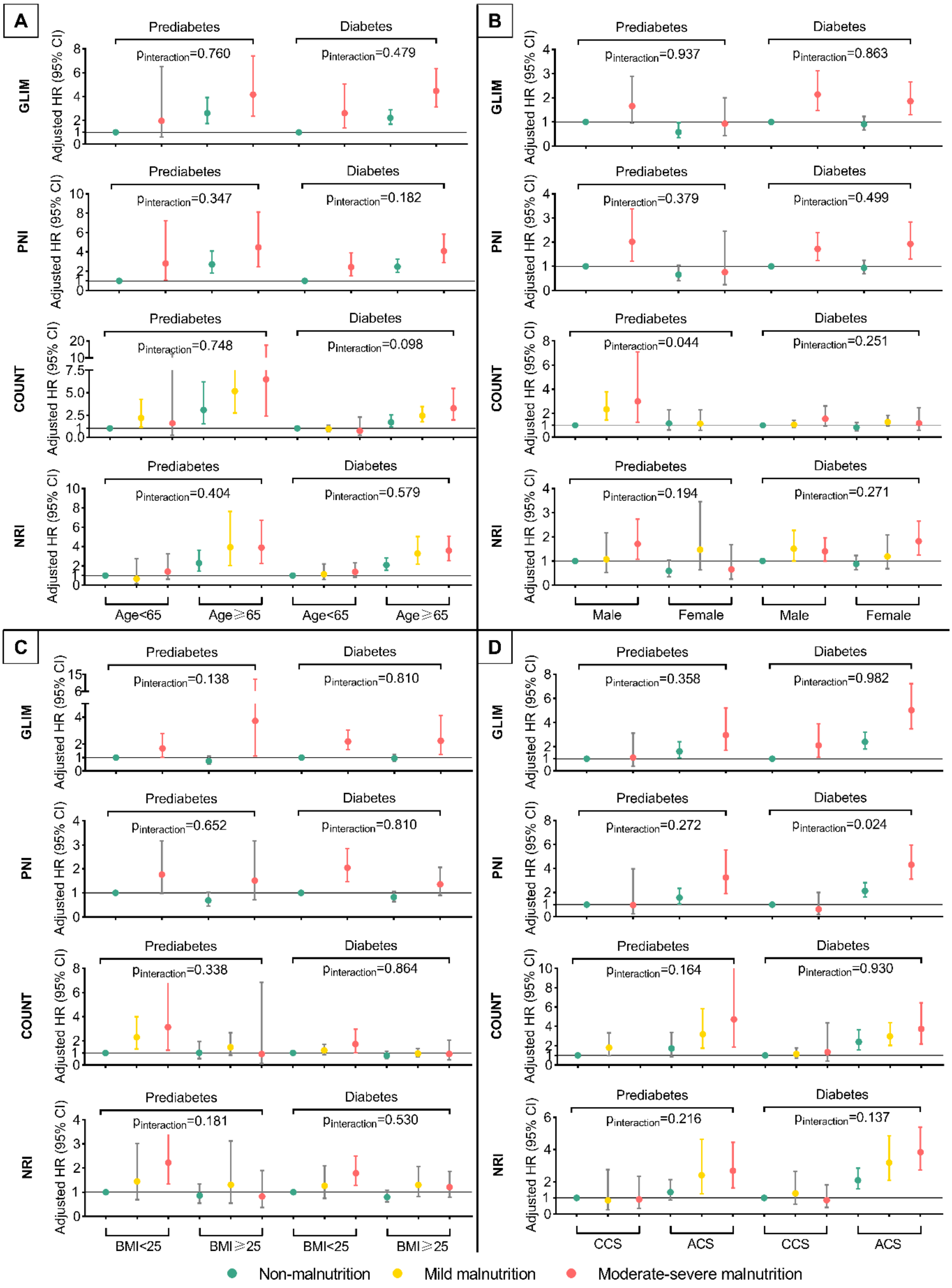

Figure S5 Subgroup analysis of associations of nutritional status with all-cause death
(A) Age; **(B)** Sex; **(C)** BMI; **(D)** presentation of coronary artery disease.
ACS, acute coronary syndrome; BMI, body mass index; CCS, chronic coronary syndrome; CI, confidence interval; COUNT, Controlling Nutritional Status; GLIM, Global Leadership Initiative on Malnutrition; HR, hazard ratio; NRI, nutritional risk index; PNI, prognostic nutritional index.

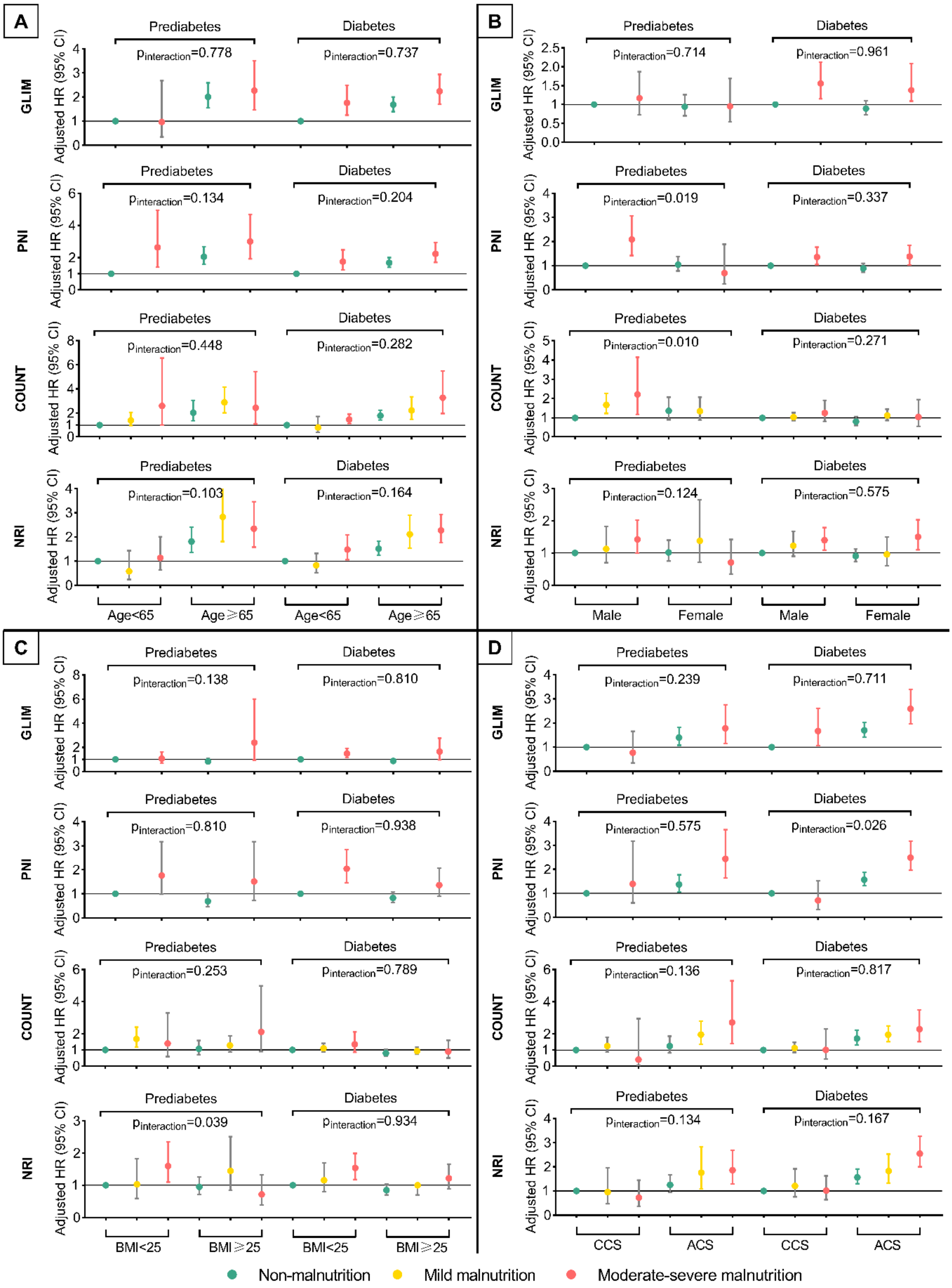

Figure S6 Subgroup analysis of associations of nutritional status with MACCEs
(A) Age; **(B)** Sex; **(C)** BMI; **(D)** presentation of coronary artery disease.
ACS, acute coronary syndrome; BMI, body mass index; CCS, chronic coronary syndrome; CI, confidence interval; COUNT, Controlling Nutritional Status; GLIM, Global Leadership Initiative on Malnutrition; HR, hazard ratio; MACCE, major adverse cardiovascular and cerebrovascular event; NRI, nutritional risk index; PNI, prognostic nutritional index.

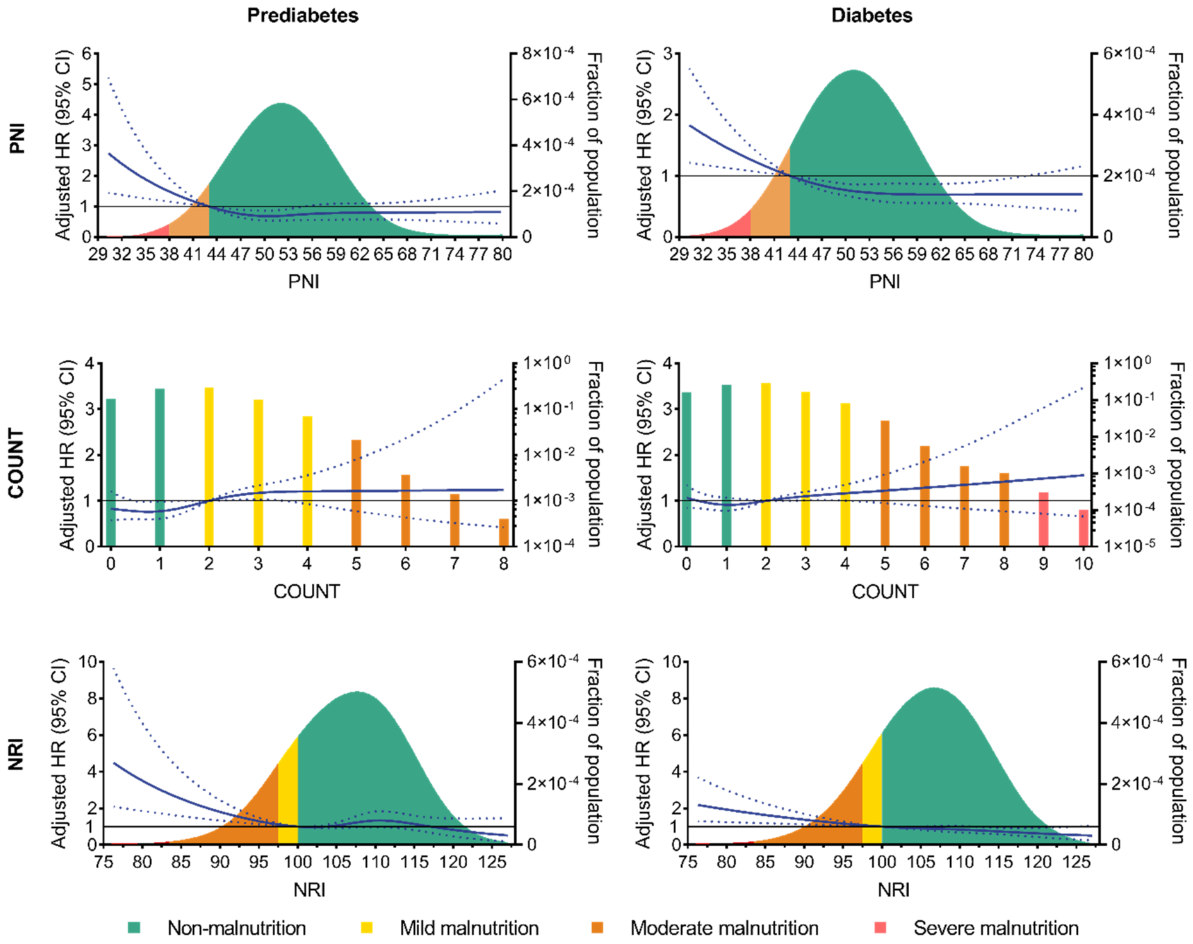

Figure S7 RCSs of PNI, COUNT and NRI for predicting MACCEs
CI, confidence interval; COUNT, Controlling Nutritional Status; GLIM, Global Leadership Initiative on Malnutrition; HR, hazard ratio; MACCE, major adverse cardiovascular and cerebrovascular event; NRI, nutritional risk index; PNI, prognostic nutritional index; RCS, restricted cubic spline.
